# Assessment of asthma control using asthma control test in chest clinics in Cameroon: a cross-sectional study

**DOI:** 10.11604/pamj.2016.23.70.8434

**Published:** 2016-03-09

**Authors:** Mbatchou Ngahane Bertrand Hugo, Pefura-Yone Eric Walter, Mama Maïmouna, Nganda Motto Malea, Olinga Ubald, Wandji Adeline, Tengang Bruno, Nyankiyé Emmanuel, Afane Ze Emmanuel, Kuaban Christopher

**Affiliations:** 1 Department of Internal Medicine, Douala general Hospital, Douala, Cameroon; 2 Faculty of Medicine and Pharmaceutical sciences, University of Douala, Cameroon; 3 Jamot Hospital, Yaounde, Cameroon; 4 Centre of Respiratory Diseases, Douala, Cameroon; 5 Office of Pneumology, Akwa, Cameroon; 6 Faculty of Health Sciences, University of Bamenda, Cameroon

**Keywords:** Asthma treatment, allergy, prevalence, Africa

## Abstract

**Introduction:**

The goal of asthma treatment is to obtain and maintain a good control of symptoms. Investigating factors associated with inadequately control asthma could help in strategies to improve asthma control. This study aimed to determine the prevalence and factors associated with inadequately controlled asthma in asthma patients under chest specialist care.

**Methods:**

A cross-sectional study was conducted from November 2012 to May 2013. Physician-diagnosed asthma patients aged 12 years and above were included. A questionnaire was used to collect demographic data, comorbidities, and medical history of asthma. Asthma control was assessed using the Asthma Control Test (ACT), with a score less than 20 for inadequately controlled asthma and a score greater or equal to 20 for controlled asthma. A multivariate analysis was used to identify factors associated with inadequately controlled asthma.

**Results:**

Overall, 243 patients were included in this study. Asthma was controlled in 141 patients (58%) and inadequately controlled in 102 (42%). The mean duration of asthma was 8 years with an interquartile range of 4 and 18 years. Forty-three participants (17.7%) were not under any controller medication while the mean ACT score was 19.3 ± 4.6. Independent associations were found between inadequately controlled asthma and female gender (OR 1.91; 95% CI 1.06-3.47) and obesity (OR 1.81; 1.01-3.27).

**Conclusion:**

Asthma remains poorly controlled in a large proportion of asthma patients under specialist care in Cameroon. Educational programs for asthma patients targeting women and based on weight loss for obese patients may help in improving the control of asthma.

## Introduction

Asthma is a common, chronic respiratory disease characterized by variable symptoms of wheeze, shortness of breath, chest tightness and/or cough, and by variable expiratory airflow limitation [1]. Worldwide, about 300 million individuals are affected by asthma [2]. It is a major health concern in developed countries as well as in low-income countries. The number of disability-adjusted life years lost due to asthma worldwide has been estimated at about 15 million per year currently and is similar to that for diabetes, liver cirrhosis, or schizophrenia [2]. The results of the International Study of Asthma and Allergies in Childhood show that the prevalence of asthma in Africa ranges from 4.4% to 21.5% with a prevalence of 5.7% in Cameroon [3]. The goal of asthma management is to achieve good control of symptoms and maintain normal activity levels [1]. Despite the progress made over the past 30 years in terms of pathophysiology and management of asthma, studies show that this condition remains largely uncontrolled. The level of asthma control is the extent to which the manifestations of asthma can be observed in the patient, or have been reduced by the treatment [4, 5]. According to the Global Initiative for Asthma (GINA), asthma control is based on the frequency of symptoms, any night waking due to asthma or limitation of activity and frequency of reliever medication use [1]. Several asthma control tools have been developed for children and adults. Among these tools is the Asthma Control Test (ACT), a simple score, not using lung function test results, which has been validated for the assessment of asthma control especially in limited-resources settings [6, 7].

A recent survey conducted in 11 European countries and involving 8000 asthma patients found that asthma was uncontrolled in 45% [8]. In the United States, a study by Schatz et al found that 51.6% of asthma patients had uncontrolled asthma [9]. In Africa, studies focused on asthma control are scarce. The AIRMAG study which involved 3 countries of North Africa reported 71.3% of uncontrolled asthma [10], whereas in another study in Ethiopia, the proportion was 71.4% [11]. Failure of asthma control increases the morbidity of the disease, affects the quality of life of patients and significantly increases the use of health care services [12, 13], and therefore generating high health care expenditures as demonstrated by Barnes et al [14]. The aim of the present study was to determine the prevalence of asthma control and factors associated with inadequately controlled asthma in asthma patients under chest specialist care in Cameroon.

## Methods

### Design and setting

A cross-sectional study was conducted from 1^st^ November 2012 to 31^st^ May 2013 in 4 chest consultations in Yaounde and Douala, the two largest cities in Cameroon. In Yaounde, patients were recruited at Jamot Hospital which is the main public chest clinic of the town. In Douala, patients were recruited in Douala General Hospital which is a tertiary care hospital and in two private chest clinics (Centre des maladies respiratoires and Cabinet de pneumologie d'Akwa).

### Study participants

Subjects included in this study were asthma patients aged 12 years or above who have been followed by a chest specialist for at least 3 months. The diagnosis of asthma was confirmed by lung function tests. Patients with heart failure, lung infection and other chronic respiratory diseases such as bronchiectasis, lung tumor and chronic obstructive pulmonary disease were excluded from the study.

### Data collection and variables

Eligible participants were identified using consultation registers at different sites of the study. They were then invited through a telephone call to participate in the study. On the arrival at the study site, they were given information about the study and a verbal informed consent was obtained before recruitment. Data were collected using a pretested questionnaire which was filled during a face-to-face interview conducted by a trained member of the study staff. Data were collected on socio-demographic characteristics (age, gender, level of education, medical insurance status) and on the presence or absence of comorbidities (allergic rhinitis, gastro-esophageal reflux disease and obesity). Obesity was defined by a body mass index (BMI) equal to or greater than 30 kg/m^2^. Smoking status and alcohol consumption were also recorded. The medical history of asthma (duration of disease, hospitalization for exacerbation, use of controller medication, chest specialist visits in the year), adherence to controller medication and asthma control were also recorded. Before the assessment of medication adherence, we verified the prescription of controller medication in patients’ records. Adherence to treatment was assessed using the eight-item Morisky Medication Adherence Scale (MMAS-8) [15, 16]. This patient-report measurement of treatment adherence has a maximum score of 8 points and is classified into 3 levels: low adherence (score < 6 points), medium adherence (score between 6 and 7 points) and high adherence (score = 8 points). The level of asthma control was measured using the Asthma Control Test (ACT) [6]. The ACT questionnaire assesses the symptoms of asthma in the last 4 weeks. Patients are asked to rate the following items: daily activity limitations, shortness of breath, nocturnal awakening and the use of rescue medications. They are also asked to rate their asthma control. Each questionnaire with an ACT score ≥20 indicates controlled asthma, while 16 to 19 indicates partly controlled asthma and ≤15 characterizes poorly controlled asthma. In the current study, we considered patients with a score < 20 as having inadequately controlled asthma.

### Data analysis

Statistical analysis was performed using IBM SPSS statistics Version 20 (Armonk, NY: IBM Corp). Categorical variables were summarized as frequencies and percentages while continuous data were described using measures of central tendency and dispersion (mean, median, standard deviation, interquartile range) as appropriate. While studying the factors associated with asthma control, inadequately controlled asthma (ACT < 20) was compared to controlled asthma (ACT ≥20). The association between potential factors and inadequately controlled asthma was explored using univariate logistic regression. All the factors that showed a p-value less than (or equal) to 0.20 were assessed in a multivariate logistic regression model using a stepwise strategy in order to identify independent factors associated with inadequately controlled asthma. Odds ratios (OR) and their 95% confidence intervals (CI) were determined. A p-value less than 0.05 was regarded as statistically significant.

### Ethical issue

The study design was in accordance with the principles of the Declaration of Helsinki. The study protocol was submitted to Cameroon's National Ethics Committee and ethical clearance was obtained. Verbal informed consent was provided by each patient before recruitment.

## Results

Overall, 243 asthma patients were included in this study. Of these patients, 81 (33.3%) were male while 162 (66.7%) were female, giving a male to female ratio of 1:2. The mean age of participants was 40.4 ± 18.5 years (range: 12-87 years). The mean duration of asthma was 8 years with an interquartile range (IQR) of 4 and 18 years. Sixty-six patients (27.2%) were covered by medical insurance whereas 177 (72.8) were not. Obesity and overweight were found respectively in 78 (32.1%) and 65 (26.7%) patients. Forty-three participants (17.7%) were not under any controller medication while the mean ACT score was 19.3 ± 4.6. The other characteristics of the study population are shown in Table 1. The prevalence of controlled asthma in this study was 58% (95% CI 51.78-64.27). Asthma was partially controlled in 57 (23.5%) patients and uncontrolled in 45 (18.5%) patients. In total, 102 (42%) patients of the study population had inadequately controlled asthma. The results of the univariate analysis (Table 2) showed that female sex, obesity and asthma related hospital admission for the past 12 months were significantly associated with inadequately controlled asthma. After adjusting for other potential factors in the multivariate regression analysis (Table 3), only female sex and obesity (OR: 1.81; 95% CI: 1.01-3.27) were independently associated with inadequately controlled asthma (OR 1.91; 95% CI 1.06-3.47).

**Table 1:** Baseline characteristics of asthma patients in chest clinics in Cameroon (N = 243)

Variables	Value	Percentage
**Gender**		
Male	81	33.3%
Female	162	67.7%
**Age: mean ± SD, years**	40.4 ± 18.5	-
**Duration of asthma (IQR), years**	8 (4 – 18)	-
**School education**		
≤ Primary school	41	16.9%
≥ secondary level	202	83.1%
**Medical insurance**		
Yes	66	27.2%
No	177	72.8%
**Smoking status**		
Current smoker	2	0.8%
Ex-smoker	9	3.7%
Non smoker	232	95.5%
**Body Mass Index**		
Normal/underweight	100	41.2%
Overweight	65	26.7%
Obese	78	32.1%
**Last hospitalization**		
< 12 months	31	12.8%
≥ 12 months	212	87.2%
**Chest physician visit**		
≤ 12 months	149	61.3%
> 12 months	94	38.7%
**GERD^a^**		
Yes	36	14.8%
No	207	85.2%
**Allergic rhinitis**		
Yes	151	62.1%
No	92	37.9%
**Controller medication**		
No	42	17.3%
Yes	201	82.7%
**Adherence to controller medication**		
Low adherence	90	44.8%
Medium adherence	73	36.3%
High adherence	38	18.9%
**ACT**		
< 20	102	42%
≥ 20	141	58%

a GERD: Gastro-oesophageal reflux disease

**Table 2:** Univariate analysis of factors associated with asthma control (N=243)

Variables	Uncontrolled asthma		OR (95% CI)	P value
	Yes (n=102)	No (n=141)		
**Age, per year increase**	-		0.99 (0.98 – 1)	0.46
**Gender**				
Female	77 (47.5%)	85 (52.5%)	2.02 (1.15 – 3.56)	0.01
Male	25 (30.9%)	56 (69.1%)		
**Level of education**				
≤ Primary school	17 (41.5%)	24 (58.5%)	0.97 (0.49 – 1.92)	0.94
≥ secondary level	85 (42.1%)	117 (57.9%)		
**Medical insurance**				
Yes	27 (40.9%)	39 (59.1%)	1.06 (0.59 – 1.88)	0.83
No	75 (42.4%)	102 (57.6%)		
**Last hospitalization**				
< 12 months	18 (58.1%)	13 (41.9%)	2.09 (0.97 – 4.49)	0.05
≥ 12 months	84 (39.8%)	128 (60.2%)		
**Chest physician visit**				
≤ 12 months	60 (40.3%)	89 (59.7%)	0.83 (0.49 – 1.40)	0.49
> 12 months	42 (44.7%)	52 (55.3%)		
**Asthma duration**				
≤ 8 years	44 (36.1%)	78 (63.9%)	1 (0.98 – 1.02)	0.61
> 8 years	58 (47.9%)	63 (52.1%)		
**Pet animals**				
Yes	32 (39%)	50 (61%)	0.83 (0.48 – 1.43)	0.50
No	70 (43.5%)	91 (56.5%)		
**Obesity (BMI ≥ 30)**				
Yes	28 (35.9%)	50 (64.1%)	0.68 (0.37 – 1.13)	0.18
No	74 (44.8%)	91 (55.2%)		
**Allergic rhinitis**				
Yes	67 (44.4%)	84 (55.6%)	1.3 (0.76 – 2.2)	0.33
No	35 (38%)	57 (62%)		
**GERD**				
Yes	19 (52.8%)	17 (47.2%)	1.69 (0.83 – 3.44)	0.14
No	82 (39.8%)	124 (60.2%)		
**Use of controller therapy**				
Yes	83 (41.3%)	118 (58.7%)	1.17 (0.6-2.29)	0.63
No	19 (45.2%)	23 (54.8%)		
**Adherence to treatment**				
Low adherence	40 (44.4%)	50 (55.6%)	0.9 (0.42 - 1.95)	0.8
Medium adherence	27 (37%)	46 (63%)	1.23 (0.55 – 2.75)	0.6
High adherence	16 (42.1%)	22 (57.9%)		

**Table 3:** Multivariate analysis of factors associated with asthma control (N = 243)

Variables	Ajusted OR (95% CI)	P-value
Female gender	1.91 (1.06 – 3.47)	0.03
Last hospitalization	2.03 (0.92 – 4.46)	0.07
Obesity (BMI ≥ 30)	1.81 (1.01 – 3.27)	0.04
GERD	1.61 (0.76 – 3.39)	0.21

## Discussion

This study is one of the few studies assessing asthma control in sub-Saharan Africa. A high proportion of patients had inadequately controlled asthma and the factors associated with inadequately controlled asthma were female sex and obesity. In a study conducted in North Africa involving asthma patients receiving both specialist and primary care, asthma was uncontrolled (ACT ≤19) in 71.3% of patients [10]. Using the same assessment tool, Zemedkun et al in Ethiopia found an uncontrolled asthma rate of 71.4% [11]. Similar results were observed by Laforest et al in a community pharmacy-based survey in France [17]. These studies were conducted in primary care settings and they demonstrate how the real-life condition of asthma patients is far from the total control of asthma which is the main objective of asthma treatment. The results of our study show a better control of asthma with 58% of patients having controlled asthma and 42% of patients with uncontrolled asthma. This finding is consistent with the results of previous studies conducted in specialist consultations [18, 19]. In fact, our patients were recruited in chest clinics and it has been demonstrated that asthma patients seen by specialists are more likely to be well managed than those followed-up by non-specialists [20, 21]. In accordance with some previous research [20, 22], we found in this study that women were more likely to have their asthma inadequately controlled. It has also been shown that asthma is usually more severe in women [23, 24], On the contrary, other reports did not find any relationship between sex and asthma control [9, 25], More research is needed to clarify the place of the female sex as a factor related to asthma control.

Another factor independently associated with inadequately controlled asthma in this study was obesity. Although few studies did not find any link between asthma control and obesity [26, 27], most of the studies show that it is a risk factor for asthma control regardless of airway inflammation, lung function and airway hyper-responsiveness [28, 29]. The pathophysiology underlying poor asthma control in obese patients is still under investigation, but current research is focusing on the role of oxidative stress and the effects of adipokines on airways inflammation [28], Because of the very low prevalence of smoking in the present study, we could not investigate its effects on asthma control. The other parameters such as age, school education, and gastroesophagal reflux disease, use of asthma controller therapy, hospitalization for exacerbation, treatment adherence and allergic rhinitis were not related to asthma control. Similar results were found in other sub-Saharan settings [11, 30]. Other studies with larger sample size are needed in sub-Saharan Africa to confirm or refute our findings. Our study has several limitations: the inhalation technique of dry powders was not assessed and adherence to treatment was based on patients’ reports. In addition, lung function tests were not performed during the assessment of asthma control. However, the asthma control test that we used has been validated and is correlated with the level of asthma control. Another limitation of this study is the underpowered sample size which might have contributed to the limited number of factors associated with asthma control.

## Conclusion

This study demonstrates that despite specialist care, a large proportion of asthmatic patients have inadequately controlled asthma in Cameroon. Educational programs targeted on women and weight loss in obese asthma patients could improve the control of asthma in our setting. Further research is needed in sub-Saharan Africa confirm our findings.

### What is known about this topic

- Asthma remains largely uncontrolled worldwide
- Studies on asthma control in sub-Saharan Africa are scarce

### What this study adds

- We found a high prevalence of inadequately controlled asthma among patients under chest specialist care
- Obesity and female sex are the main associated factors for inadequately controlled asthma
